# A Novel Electrochemical Sensing Strategy Based on Poly (3, 4-ethylenedioxythiophene): Polystyrene Sulfonate, AuNPs, and Ag^+^ for Highly Sensitive Detection of Alkaline Phosphatase

**DOI:** 10.3390/nano12193392

**Published:** 2022-09-28

**Authors:** Jiangshan Lei, Jian Kang, Jifa Liu, Guannan Wang

**Affiliations:** 1 College of Pharmacy, Jinzhou Medical University, Jinzhou 121001, China; 2 College of Biomedical Engineering and the Key Laboratory for Medical Functional Nanomaterials, Jining Medical University, Jining 272067, China

**Keywords:** electrochemical immunosensor, alkaline phosphatase (ALP), poly (3, 4-ethylenedioxythiophene): polystyrene sulfonate (PEDOT:PSS), gold nanoparticles(AuNPs), silver

## Abstract

Alkaline phosphatase (ALP) is a crucial marker for the clinical analysis and detection of many diseases. In this study, an accurate signal amplification strategy was proposed for the sensing and quantification of alkaline phosphatase using poly (3, 4-ethylenedioxythiophene):polystyrene sulfonate (PEDOT:PSS), gold nanoparticles (AuNPs), and Ag^+^. Signal amplification was achieved by the modification of PEDOT:PSS and AuNPs on glassy carbon electrodes. Atomic force microscopy was performed to characterize the morphology of the modified nanomaterials. To detect ALP, 1-naphthyl phosphate (1-NP) was used as the substrate, and alkaline phosphatase catalyzed 1-NP into 1-naphthol (1-N), which resulted in the reduction of Ag^+^ to Ag^0^ on the surface of the modified electrode (AuNPs/PEDOT:PSS/GCE). The deposition of Ag drastically enhanced the detection signal. Differential pulse voltammograms of 1-N, which is the enzymatic product from the ALP reaction with 1-NP, were recorded. In the linear range of 0.1–120 U L^−1^, a quantitative analysis of alkaline phosphatase was achieved, with high sensitivity and a low detection limit of 0.03 U L^−1^. Stable, selective, and reproducible electrochemical sensors were designed. Moreover, the proposed electrochemical sensor exhibited a prominent sensing performance in the spiked diluted human serum. Thus, the sensor can be used in numerous applications in alkaline phosphatase or other analyte detection.

## 1. Introduction

Alkaline phosphatase (ALP) is a phosphate lyase with a wide range of substrate specificity, and it can be used to catalyze the hydrolysis of numerous phosphorylated compounds [1]. In clinical practice, ALP is a critical enzyme in various tissues of the human body and participates in a range of physiological functions in biological systems [2]. The ALP produced by various tissues differs considerably depending on the health of the individual [3]. Therefore, ALP is a critical indicator for the diagnosis of many diseases, including bone disease, hepatobiliary dysfunction, breast cancer, and prostate cancer [4,5,6,7]. The development of a high-sensitivity ALP quantitative technique is critical for clinical diagnosis and related biological research. The quantitative detection of alkaline phosphatase has been advanced by many analytical methods, such as electrochemiluminescence [1], fluorescence [8], colorimetric [9] surface-enhanced Raman scattering (SERS) [10], and electrochemical immunoassay [6]. Among the various analytical methods, electrochemical analytical methods exhibit superior results.

Electrochemical analysis methods can be used in numerous applications because of their unique properties, including portability, high sensitivity, simplicity, low maintenance costs, and time-saving characteristics [11,12,13,14,15]. Considerable progress has been achieved in improving detection sensitivity [16]. Various signal amplification strategies have been proposed for alkaline phosphatase detection. For example, Zhu et al. designed a signal-amplified electrochemical analysis method based on ring-opening polymerization (ROP) [17]. Wang et al. investigated a signal amplification strategy using aminoferrocene (AFC) as an electroactive probe coupled with single-stranded DNA (ssDNA) [18]. Despite the low detection limits of these studies, challenges such as high cost, complex processes, and long experimental time remain. The collaboration of nanomaterials with effective electrochemical analytical techniques has attracted considerable research attention in biosensors. The electrically conducting polymer poly (3, 4-ethylenedioxythiophene):polystyrenesulfonate (PEDOT:PSS) is a highly promising nanomaterial. PEDOT combines with negatively charged poly (styrenesulfonic acid) (PSS) to form a water-based complex (PEDOT:PSS) [19]. Because of its high conductivity and excellent environmental stability, PEDOT:PSS is one of the most attractive electronic materials for solar cells and supercapacitors [20,21]. Excellent flexibility and easy processability render PEDOT:PSS an excellent candidate for the fabrication of electrode modification materials [22].

With the rapid development of nanotechnology, noble metal nanoparticles, as versatile nanomaterials, have attracted considerable research attention and play a crucial role in biomedical fields [23]. In particular, gold nanoparticles (AuNPs) exhibit excellent electrical conductivity, a large surface area, high loading capacity, and superior biocompatibility, which make AuNPs excellent candidates for noble metal nanoparticles [24,25]. Because of their unique characteristics, AuNPs have been widely used in electrochemical sensors to enhance electrochemical signal transduction and lower the detection limit. Because preparation of AuNPs by chemical reduction is highly polluting, the development of an environmentally friendly method to prepare Au nanoparticles through electrochemistry is critical [26,27].

**Scheme 1:**
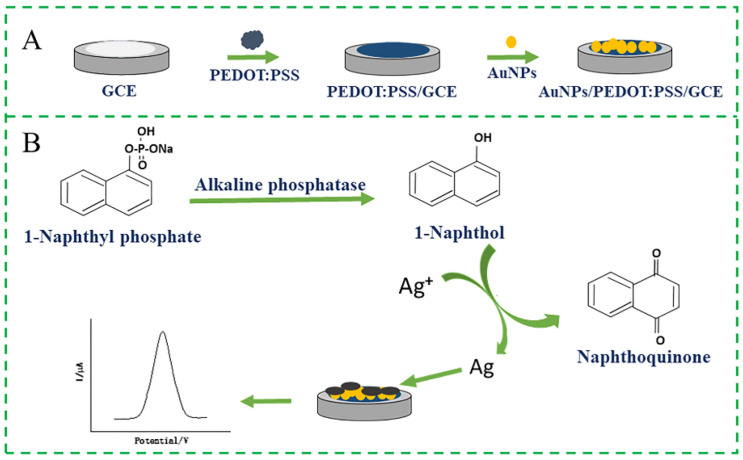
Schematic of the sensing strategy for ALP detection: (**A**) the preparation of the electrochemical electrode with PEDOT:PSS and AuNPs; (**B**) the electrochemical detection principle of ALP.

In this study, a simple and ultrasensitive electrochemical sensing strategy based on PEDOT:PSS, AuNPs, and Ag^+^ was proposed for ALP detection. As presented in Scheme 1, the detection principle of ALP is based on ALP catalyzing 1-NP into 1-N, then the oxidation of 1-N reduces Ag^+^ to Ag^0^ on the surface of the AuNPs, and the electrochemical signals of the oxidation product naphthoquinone is recorded. An efficient amplification platform was designed by using the conductive polymer PEDOT:PSS and AuNPs as the electrochemical sensor electrode modifier. The decoration of the PEDOT:PSS and AuNPs can drastically enhance electron transfer efficiency and improve the sensitivity of electrochemical sensing. Crucially, AuNPs have been used as the seed of silver deposition, which considerably improves the metallization reaction induced by the enzyme and avoids ALP inactivation because of Ag deposition. Furthermore, Ag deposition on the surface of AuNPs plays a crucial role in enhancing the electrochemical response. To the best of our knowledge, the PEDOT:PSS and AuNP composite is yet to be used for ALP detection. Therefore, this study was the first to use this composite for ALP detection. The proposed method is simple and ultrasensitive, and suitable for application and promotion in electrochemical analysis.

## 2. Experiment

### 2.1. Apparatus

CV, differential pulse voltammetry (DPV), and EIS were performed using a CHI650E electrochemical workstation (Shanghai CH Instrument Company, Shanghai, China). A conventional three-electrode system was used in this study. A glassy carbon electrode (GCE), platinum wire electrode, and saturated Ag/AgCl (KCl) were used as the working electrode, the counter electrode, and the reference electrode, respectively. An FM-Nan oview6800 microscope was used to perform atomic force microscopy (AFM) (FEISHIMAN, Suzhou, China).

### 2.2. Reagents

PEDOT:PSS (Clevios PH1000) was purchased from Heraeus, Germany. The surfactant PEG-TmDD was purchased from Tianjin SurfyChem t&d Co., Ltd. (Tianjin, China). ALP, 1-naphthalene phosphate sodium, 1-naphthol, chloroauric acid (HAuCl_4_), glucose oxidase (GO_X_), ethylene glycol, potassium ferricyanide (K_3_Fe(CN)_6_), and potassium ferrocyanide (K_4_Fe(CN)_6_) were purchased from Sigma-Aldrich Ltd. (St. Louis, MO, USA). Bovine serum albumin (BSA) and p53 protein were purchased from Abcam Ltd. (Boston, MA, USA). Human serum samples were obtained from the Affiliated Hospital of Jinzhou Medical University. Potassium chloride (KCl) and silver nitrate were purchased from Sinopharm Chemical Reagent Co., Ltd. (Beijing, China). Phosphate-buffered saline (PBS) was obtained from sodium dihydrogen phosphate (NaH_2_PO_4_) and disodium hydrogen phosphate (Na_2_HPO_4_) and adjusted to various pH levels using sodium hydroxide (NaOH) and phosphoric acid (H_3_PO_4_). Double-distilled water was used during the experimental analysis.

### 2.3. Construction of the Electrochemical Sensor

High-conductivity PEDOT:PSS was synthesized according to our previous report [28]. Prior to decoration, the GCE was carefully polished with 0.3 and 0.05 μm alumina powder slurries, and cleaned with pure water. The GCE was ultrasonically cleaned in ethanol and pure water to obtain a smooth mirror-like surface, and it was allowed to dry under room conditions in nitrogen. Next, 10 μL of the as-prepared PEDOT:PSS solution was dropped onto the surface of the fresh GCE electrode with a pipette, and the modified electrode was dried naturally at room temperature. PEDOT:PSS exists on the surface of the GCE as a uniform film. Subsequently, AuNPs were electrically deposited on the surface of the modified electrode (PEDOT:PSS/GCE) in an environmentally friendly manner. The modified electrode (PEDOT:PSS/GCE) was immersed in a 0.5 mM HAuCl_4_ solution containing 0.1 M KCl and subjected to a potential sweep between −0.8 and 1.5 V for 15 cycles [29,30]. After AuNP electrodeposition, the obtained modified electrodes were denoted as AuNPs/PEDOT:PSS/GCE. The AuNPs/PEDOT:PSS/GCE were washed slowly with PBS (pH 7.5), and finally dried at room temperature.

### 2.4. Electrochemical Detection of ALP

For ALP detection, various units of ALP were added into the PBS (pH 9) containing 1-naphthalene sodium phosphate at a concentration of 4 mg mL^−1^ and AgNO_3_ at a concentration of 1 mM. Next, the pre-prepared AuNPs/PEDOT:PSS/GCE was immersed in the detection solution immediately for incubation. The generated current signals were detected in PBS containing 0.1 M KCl by a CHI660E electrochemical workstation after incubation for 4 min. DPV was performed with a pulse width and pulse period of 0.2 and 0.5 s for electrochemical detection [3,31].

## 3. Results and Discussion

### 3.1. Morphology Characterization of the PEDOT:PSS and AuNPs/PEDOT:PSS

**Figure 1:**
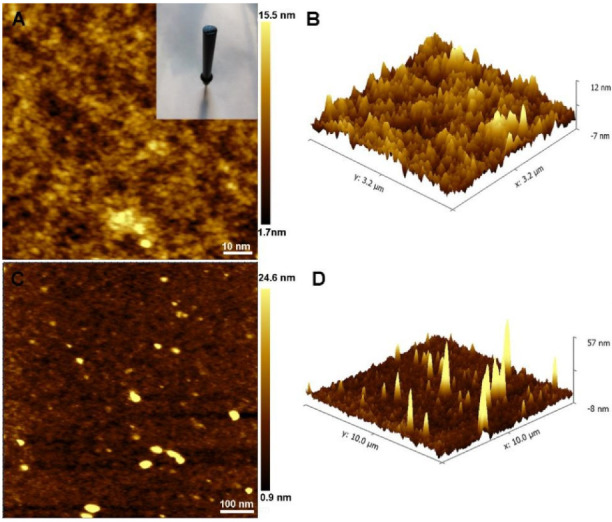
Two-dimensional planar AFM images of PEDOT:PSS (**A**), the picture inset in (**A**) displays a photograph of PEDOT:PSS modified to the GCE surface. Three-dimensional AFM images of PEDOT:PSS (**B**), two-dimensional planar AFM images of AuNPs/PEDOT:PSS (**C**), and three-dimensional AFM images of AuNPs/PEDOT:PSS (**D**).

The electron transfer capability is closely related to the morphology of the films [32]. In this work, AFM was used to perform the morphological characterization of the PEDOT:PSS and AuNPs/PEDOT:PSS. As displayed in Figure 1A, the PEDOT:PSS layer exhibited a smoother surface than the AuNPs/PEDOT:PSS layer, and 3D AFM images of the PEDOT:PSS exhibited uniform low peaks at altitudes of −7 to 12 nm, as shown in Figure 1B. As displayed in the inset of Figure 1A, PEDOT:PSS reside on the surface of GCE as uniform films. Furthermore, as displayed in Figure 1C,D, many high spikes were observed on the PEDOT:PSS surface after AuNP deposition compared with the images of the PEDOT:PSS, which resulted in an increase in the surface area and roughness of the modified electrode, which considerably improved the availability of active sites. AFM images revealed that gold nanoparticles were electrodeposited onto the surface of the modified electrode (PEDOT:PSS/GCE).

### 3.2. Electrochemical Analytical Performance of AuNPs/PEDOT:PSS

**Figure 2:**
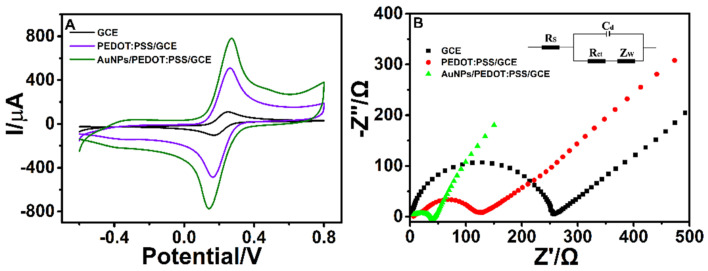
CV (**A**) and EIS (**B**) of the bare GCE, PEDOT:PSS-modified GCE, and AuNP/PEDOT:PSS-modified GCE. Inset of B: Randles equivalent circuit.

To investigate the variation of the electrochemical performance of electrochemical sensors before and after PEDOT:PSS and AuNPs/PEDOT:PSS modification, the CVs of these modified electrodes were tested in 0.01 M PBS (pH 7.5) containing 5 mM of [Fe(CN)_6_]^3–/4–^ and 0.1 M of KCl [33]. As displayed in Figure 2A, a pair of quasi-reversible redox peaks were obtained on these three electrodes in the scanning potential range of −0.6 to 0.8 V. A set of small redox peaks were observed on bare GCE because of the low electrochemical response. As displayed in CVs, when PEDOT:PSS was fixed onto the GCE electrode, the redox peak current was enhanced considerably. Thus, the PEDOT:PSS modified on GCE could satisfactorily achieve a signal amplification effect because of its excellent conductivity. Moreover, the redox peak current was increased after the modification of AuNPs. This increased signal response can be attributed to the excellent electron conductivity and high surface area of AuNPs. Particle size and shape are two important parameters that directly affect the efficiency of electrochemical sensing. AuNPs have become a metal-based nanomaterial that warrant a lot of research attention due to their small size, high surface area, and good electronic properties [34]. In this study, the Randles–Sevcik equation was used to calculate the electrochemically active area as follows: *I*_p_ = 2.69 × 10^5^AD^1/2^n^3/2^v^1/2^C, where *I*_p_ is the peak current, *n* is the number of electrons transferred in the redox reaction, *A* is the electrode area, *D* is the diffusion amount of the redox probe, *C* is the concentration of the redox probe in the electrolyte, and v is the scanning rate used in the experiment [35]. By our calculation, the electrochemical-active area of modified AuNPs increased by 47.4%. Notably, AuNPs increased the electrochemical-active region and promoted the response of the electrochemical signal.

EIS is a technique for investigating the electronic interface properties of various electrodes in 0.01 M PBS (pH 7.5), comprising 5 mM [Fe(CN)_6_]^3−/4−^ and 0.1 M KCl. As indicated in Figure 2B, a linear part and a semicircular part were observed in the Nyquist diagrams, and the Randles equivalent circuit (Figure 2B, inset) fitting the Nyquist plot is displayed [36], which includes the solution resistance (*R_s_*), the charge transfer resistance (*R_ct_*), Warburg impedance (*Z_W_*), and the capacitance (*C_d_*). The charge transfer resistance (*R_ct_*) is associated with the semicircular diameter. As displayed, the EIS of the electrodes revealed that the EIS pattern changes were similar to the trend of CV. The EIS curve of bare GCE exhibited a large semicircular diameter (257 Ω), which indicated that the electron transfer rate was the slowest. Compared with the bare GCE, a small semicircular diameter appeared after modifying PEDOT:PSS (123 Ω), because the high electroconductibility of PEDOT:PSS resulted in electron transfer. Moreover, the PEDOT:PSS was modified on the bare GCE surface. When the electrode surface was electrodeposited by AuNPs, the semicircular diameter (40 Ω) considerably reduced again because of the excellent metal conductivity and large surface area. The decreasing *R_ct_* value revealed that the purposed electrochemical sensors were successfully fabricated.

**Figure 3:**
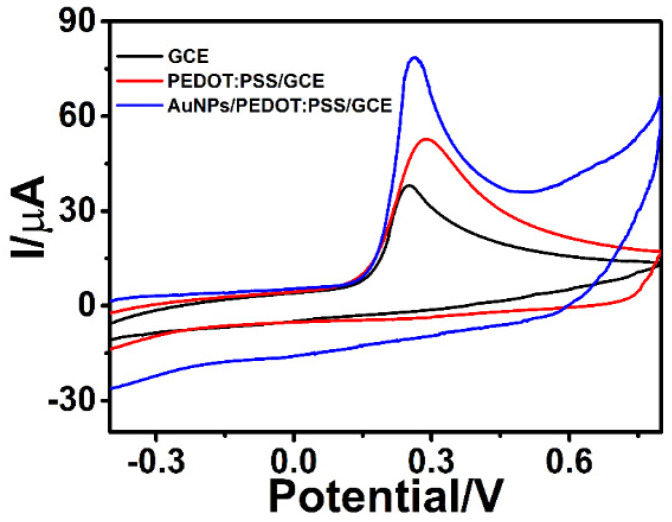
CV of the bare GCE, PEDOT:PSS-modified GCE, and AuNP/PEDOT:PSS-modified GCE in 0.01 M PBS (pH 9.0) containing 0.1 mM 1-naphtol.

To detail the potential role of AuNPs/PEDOT:PSS in signal amplification in detecting ALP, the electrochemical behavior of 1-naphthol, a hydrolysate of ALP, was investigated using CV curves. As displayed in Figure 3, the bare GCE exhibited an oxidation peak at approximately 0.26 V, and the peak current of the oxidation peak was approximately 38 μA. When the bare electrode was modified with PEDOT:PSS, the peak current of the oxidation peak increased considerably, and the peak current was approximately 53 μA. With the deposition of AuNPs on the surface of GCE, the peak current increased to approximately 79 μA, and the peak current response value was two times that of the blank GCE. The results revealed that PEDOT:PSS and AuNP nanocomposites exhibited a strong signal amplification effect. Thus, the nanocomposites exhibited potential applications in ALP detection.

**Figure 4:**
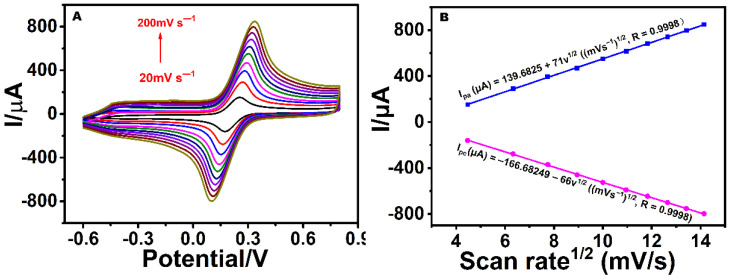
CV performance of AuNPs/PEDOT:PSS/GCE at various scan rates (20, 40, 60, 80, 100, 120, 140, 160, 180, 200 mV s^–1^) (**A**), the relationship between the square root of the scan rate and peak current (**B**).

To prove that the redox reaction on the electrode was a diffusion-controlled process, CV analysis of the constructed sensor at scan rates of 20, 40, 60, 80, 100, 120, 140, 160, 180, and 200 mV s^−1^ revealed that the anodic and cathodic peak currents increased with the increase in the scanning rate (Figure 4A) [37]. The linear dependence between the peak current and scanning rate for the same platforms was obtained using 5.0 mM [Fe(CN)_6_]^4−/3−^ solution and 0.1 M of KCl in 0.01 M PBS, pH 7.5. Regarding the quantitative relationship between the peak current and scan rate in Figure 4B, the equations of calibration curves for peak current and scanning rate were as follows: (anodic peak current) *I*_pa_ (μA) = 139.6825 + 71v^1/2^ ((mVs^−1^)^1/2^, R = 0.9998) and (cathodic peak current) *I*_pc_(μA) = −166.68249−66v^1/2^ ((mVs^−1^)^1/2^, R = 0.9998), respectively. The direct proportion of the peak current and square root of scan rate revealed that the diffusion-controlled redox reaction process on the electrode surface had an excellent linear relationship [38,39].

### 3.3. Optimization of pH and the Incubation Time

**Figure 5:**
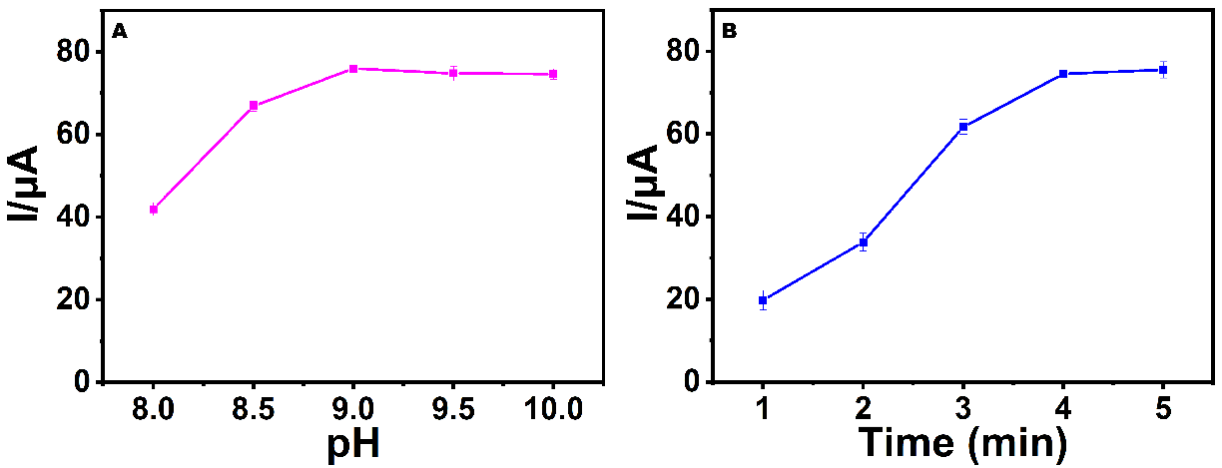
Results of optimization studies: pH of PBS (**A**) and incubation time of ALP (**B**).

The detection result of the electrochemical sensor was susceptible to many factors. Among these factors, the most critical was the pH of PBS and incubation time of ALP. ALP activity is susceptible to pH and incubation time [40,41]. To achieve the best performance of the sensing platform, the change in the peak current response measured by the electrochemical sensor in 0.01 M of PBS buffer solution with pH 8–10 is displayed in Figure 5A. The peak current response increased with the increase in the pH from 8 to 9, and reached its maximum at pH 9. According to this result, pH 9 was the optimal pH value.

The incubation time of ALP in the electrolytic cell was investigated to understand the optimal behavior of the modified electrode. As displayed in Figure 5B, the peak current response increased substantially with the increase in the accumulation time. When the incubation time reached 4 min, the peak current response tended to be stable. Therefore, the optimal incubation time was fixed at 4 min.

### 3.4. Electrochemical Determination of ALP

**Figure 6:**
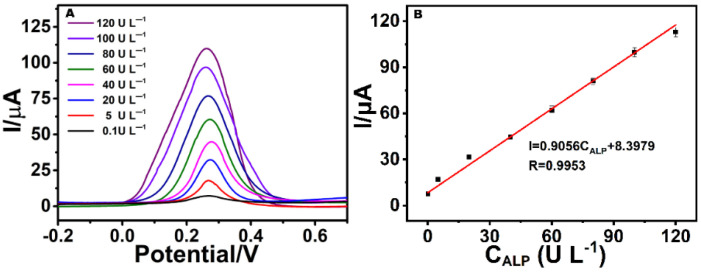
Differential pulse voltammetry (DPV) of various ALP concentrations (0.1–120 U L^–1^) for the AuNP/PEDOT:PSS-modified GCE in 0.01 M PBS (pH 9.0) containing 4 mg mL^–1^ 1-naphthyl phosphate and 1 mM AgNO_3_ (**A**). Calibration curves of the immunosensor to various concentrations of ALP (**B**); error bars represent the standard deviation.

Under optimized experimental conditions, the electrochemical sensor responses toward various concentrations of ALP were investigated in 0.01 M PBS (pH 9) containing 4 mg mL^–1^ 1-naphthalene sodium phosphate and 1 mM AgNO_3_. As displayed in Figure 6A, as the concentration of ALP increased, the peak responses of ALP increased linearly. The linear relationship existed between the peak current response of DPV and a concentration ranging from 0.1 to 120 U L^–1^. The linear regression equation can be expressed as follows: I = 8.3979 + 0.9056C_ALP_ (R = 0.9953). Crucially, the limit of detection was estimated to be 0.03 U L^–1^ according to a signal-to-noise ratio of 3. The results obtained using the proposed electrochemical sensor were compared with those previously reported in the literature in Table 1. Notably, the electrochemical sensor we designed has a wider linear range and lower detection limit than other methods used to detect alkaline phosphatase, indicating that the it has superior sensitivity. This high sensitivity could be attributed to the following two factors: first, the high conductivity and large surface area of PEDOT:PSS and AuNPs improved the sensitivity of electrochemical sensing; second, the deposition of silver again strengthened the electrical signal response.

### 3.5. Signal Amplification with Ag

**Figure 7:**
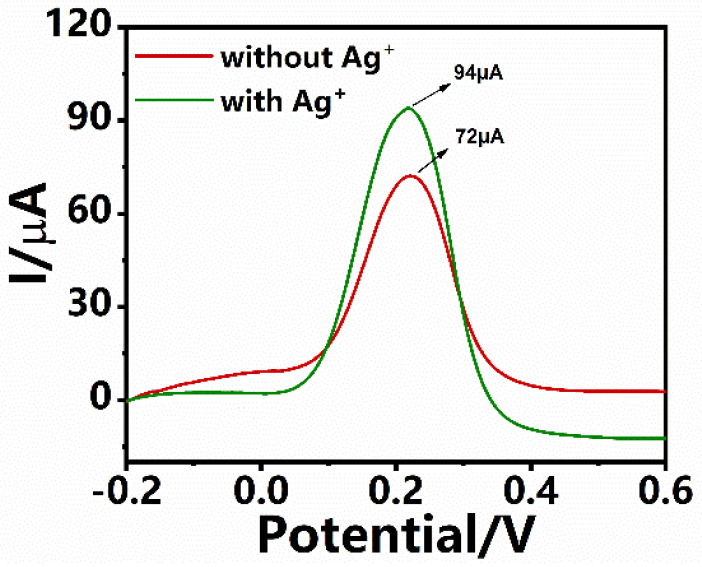
DPV current responses of the electrochemical sensor before and after treatment with Ag^+^ in 0.01M PBS (pH = 9) containing 4 mg mL^–1^ 1-naphthalene sodium phosphate.

To investigate the feasibility of signal amplification by the enzyme-induced metallization (Ag^+^ to Ag^0^) strategy, the DPV curves were recorded in the presence and absence of Ag. As displayed in Figure 7, the enzyme-induced metallization reaction did not occur in the absence of Ag. However, the current signal of the DPV was considerably amplified by the presence of PEDOT:PSS and AuNPs. The current response of DPV was amplified in the presence of Ag. Further amplification of the current response of DPV was related to enzymatic silver deposition on the surface of AuNPs/PEDOT:PSS/GCE, with gold nanoparticles as seeds.

### 3.6. Selectivity, Repeatability, and Stability of the Detection

**Figure 8:**
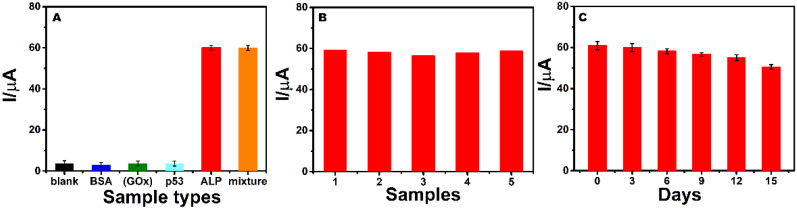
Selectivity studies of the immunosensor after incubating with blank, various interferences (100U L^−1^ BSA, 100U L^−1^ GOX, 100U L^−1^ p53, 100U L^−1^ a mixture of all), and 60U L^−1^ ALP (**A**), reproducibility studies of the immunosensor under the same experimental conditions for detecting ALP with the concentration of 60 U L^−1^ (**B**), storage stability studies of the immunosensor over time (**C**).

Specificity, repeatability, and stability are vital parameters for assessing the analytical performance of established strategies. In this study, we added BSA, glucose oxidase (GOX), p53 protein, ALP, and their mixtures. The results in Figure 8A validated that, compared with the blank signal, BSA, (GOX), and p53 were close to the values detected by the biosensor. However, considerable differences were observed in DPV peak and blank, BSA, (GOX), and p53 between ALP and the mixture, which could be attributed to the excellent specificity of the antibody.

Reproducibility is a crucial parameter for biosensor assessment. As shown in Figure 8B, a relative standard deviation (RSD) value of peak current was 1.6%, which was obtained for five consecutive determinations. The results revealed that the values were the same and exhibited high reproducibility.

To investigate the long-term stability of the sensor, we placed the sensor in 4 °C air and measured the peak DPV current in 15 days. As shown in Figure 8C, the electrode maintained 82.7% of the original response current, which indicated that the designed electrochemistry achieved satisfactory stability.

These results firmly proved that the biosensor was constructed as expected.

### 3.7. Analytical Application

**Figure 9:**
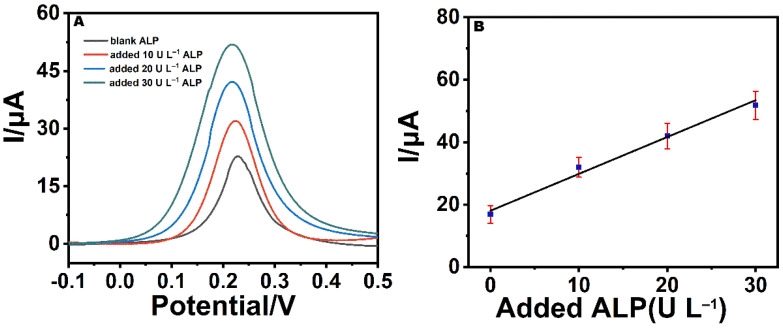
DPVs of various ALP concentrations (blank ALP, added 10 U L^–1^ALP, added 20 U L^–1^ ALP, added 30 U L^–1^ ALP) for the AuNP/PEDOT:PSS-modified GCE in human serum (pH 9.0) containing 4 mg mL^–1^ 1-naphthyl phosphate and 1 mM AgNO_3_ (**A**), and the corresponding calibration plot (**B**). Error bars represent the standard deviation.

Analytical detection after the addition of standard ALP samples to human serum was discussed for the designed method. Because serum sample substrates contain ALP, this strategy was first applied to the five-fold diluted serum samples of blank ALP detection. ALP in blank serum samples was estimated and quantified to be approximately 16.88 U L^−1^ (±2.55). In the diluted serum sample, ALP was approximately 84.42 U L^–1^ (the physiological range of ALP in normal adult serum is 20–140 U L^–1^). Subsequently, the peak current value after adding the standard ALP sample was recorded using DPV. As shown in Figure 9A, when different concentrations of alkaline phosphatase (blank ALP, added 10 U L^–1^ALP, added 20 U L^–1^ ALP, added 30 U L^–1^ ALP) were added to the diluent of human serum, the DPV responses were significantly different, and the current response gradually increased with the increase in the concentration. The linear relationship between the peak current response of DPV and the concentration of added ALP is shown in Figure 9B. All experiments complied with relevant laws and agency guidelines, and all were approved by agency committees. As presented in Table 2, after adding the standard, the spiking yields of the 10, 20, and 30 U L^–1^ALP samples were 91.4%, 101.1%, and 103.5%, respectively, and the RSD range of difference was 7.8–8.8. The results revealed that this strategy can be widely used in the determination of ALP in clinical practice.

## 4. Conclusions

In the present study, a novel electrochemical-sensing strategy based on PEDOT:PSS, AuNPs, and Ag^+^ was constructed for detecting ALP rapidly and sensitively. Because of the advantages of PEDOT:PSS and AuNPs in terms of high conductivity and a large surface area, the electron transfer efficiency of the sensor was enhanced considerably when the PEDOT:PSS and AuNPs were modified on the electrode surface, and the sensitivity could be drastically improved. Notably, an enzyme-induced metallization reaction (Ag^+^ to Ag^0^) was developed based on the amplification strategy to further enhance the electrochemical signal. The proposed method displayed a wider linear range and a lower detection limit, rendering the model an effective method for ALP detection. Therefore, this work provided a novel and promising opportunity for biomarker detection with high sensitivity and high selectivity.

## Figures and Tables

**Table 1:** Comparison of the present method with other methods for ALP detection.

Method	Linear Range (U L^–1^)	Detection Limit (U L^–1^)	Reference
Electrochemiluminescence	2–25	2	[42]
Electrochemiluminescence	5–50	0.8	[1]
Fluorescence	0.3–7.5	0.3	[43]
Electrochemical immunoassay	10–120	5.4	[44]
Fluorescent technology	0.0–1000	0.87	[45]
Fluorescent technology	0.1–1000	0.08	[46]
colorimetric methodology	0.1–1.5	0.05	[47]
Fluorescent technology	1–100	0.06	[48]
colorimetric methodology	0.8–320	0.38	[49]
Electrochemical immunoassay	0.1–120	0.03	This work

**Table 2:** Results of the calculated and known concentrations of ALP spiked in human serum (n = 5).

Sample	Added (U L^–1^)	Total Found (U L^–1^)	Recovery %	RSD %
1	10	26.02 ± 2.82	91.4	8.8
2	20	37.10 ± 3.64	101.1	8.6
3	30	47.93 ± 4.04	103.5	7.8

## Data Availability

The datasets generated during and/or analyzed during the current study are not publicly available at this time as the data also form part of an ongoing study, but are available from the corresponding author on reasonable request.
